# Supplementary material for: Response of runoff towards land use changes in the Yellow River Basin in Ningxia, China
Source: PLoS One. 2022 Apr 1;17(4):e0265931. doi: 10.1371/journal.pone.0265931 (PMC8975145; doi:10.1371/journal.pone.0265931)
Supplement: Attachment — Submitted filename: Response to Editor_R1.docx [file pone.0265931.s002.docx]

Dear Editor,

Thank you for your letter concerning my manuscript. Those comments are all valuable and very helpful for revising and improving our paper, as well as the important guiding significance to my researches. We have studied comments carefully and have made correction which I hope meet with approval. The main correction in the paper and the responds to the Journal’s comments are as flowing:

**Comment 1:** Thank you for letting us know the data for Figures 1 and 2 are from the Resource Environmental Science and Data Center of the Institute of Geographic Sciences and Natural Resources Research of Chinese Academy of Sciences.

Unfortunately, the source does not provide enough information so that we can confirm the data can be republished under a CC BY 4.0 license, which allows for unlimited reproduction including commercial use.

For this reason, we ask that you please seek permission from the data source

**Response 1:** Thank you for your comment. I have completed the Permission form and upload the granted permission to the manuscript as a Supporting Information file. In the figure caption of the copyrighted figure, include the following text: “Republished from [ref] under a CC BY license, with permission from [name of publisher], original copyright [original copyright year],” and re-uploaded the revised manuscript.

**Comment 2:** We note that several of your files are duplicated on your submission. Please remove any unnecessary or old files from your revision, and make sure that only those relevant to the current version of the manuscript are included.

**Response 2:** Thank you for your comment. I have removed unnecessary or old files from my revision.

**Comment 3:** We notice that your manuscript file was uploaded on May 21, 2021. Please can you upload the latest version of your revised manuscript as the main article file, ensuring that does not contain any tracked changes or highlighting.

**Response 3:** Thank you for your comment. I can not remove my manuscpipt file that uploaded on May 21, 2021, so I re-uploaded the latest version of my revised manuscript.

**Funding:** The research has obtained the support from the Priority Research and Projects for Ningxia in China (2020BEG03021) , the First-class Major Foundation of Ningxia Institutions of High Education in China (NXYLXK2021A03)，Natural Science Foundation of Ningxia Province (2020AAC03058, 2021AAC02007), the First-class Discipline Construction Project in Ningxia Universities: Mathematics, and the Scientific Research Project of Ningxia Colleges and Universities, China(NGY2020006).
